# Evolutionary game on mutually influenceing double-layer network

**DOI:** 10.1371/journal.pone.0317923

**Published:** 2025-01-31

**Authors:** Qinzhi Hao, Haochun Yang, Yao Sun, Tao Xu, Huang Huang

**Affiliations:** 1 Air Force Engineering University, Xi’an, China; 2 School of Computer Science and Engineering, Northwestern Polytechnical University, Xi’an, China; University of Electronic Science and Technology of China, CHINA

## Abstract

In recent years, coupled double-layer networks have played an increasingly critical role in evolutionary game theory. Research indicates that these networks more accurately reflect real-world relationships between individuals. However, current studies mainly focus on unidirectional influence within double-layer networks. Based on this, we propose a strongly coupled double-layer network cooperation evolution model. Strength individuals are located in the upper network layer, influencing the strategy choices of ordinary individuals in the lower layer, and vice versa. Monte Carlo simulations show that strength individuals can effectively enhance overall group cooperation. Under low temptation to defect, the group maintains a high cooperation rate; under high temptation, the presence of strength individuals prevents the group from falling into total defection, helping ordinary individuals escape the defection dilemma and improve cooperation levels.

## 1. Introduction

Evolutionary game theory is a branch of game theory that combines evolutionary biology and economics to study how strategies evolve over time within a population. Unlike classical game theory, the individuals in evolutionary game theory are boundedly rational. This means they cannot achieve strategic equilibrium through a single choice but must continuously learn and adjust their strategies [1]. In the context of a dilemma model that meets certain conditions, participants are assigned as research subjects and given a fixed set of strategies. After setting the initial conditions, all participants in each round engage in strategy interactions with their opponents to obtain corresponding payoffs, then update their strategies based on learning rules. Repeating this process, all individuals in the system ultimately reach a dynamic evolutionary stable equilibrium, which corresponds to the Nash equilibrium in classical game theory [2]. Common models widely applied in evolutionary game theory include the Prisoner’s Dilemma [3], Snowdrift Game [4], and Public Goods Game [5].

Complex networks, with their powerful network representation capabilities and convenient methods for measuring various network metrics, have been widely applied in network modeling. In current modeling technologies for clusters, complex network modeling is indispensable. Various complex systems exist widely in human society and the natural world, such as ecosystems, food chain systems, transportation systems, communication systems, power systems, neural systems, logistics systems, combat systems, and unmanned cluster systems. Many of these systems can be simplified into forms of nodes and edges and considered as complex networks [6], allowing for analysis and research into complex topological structures and dynamic behaviors. Examples include simplifying them into food chain networks, airline networks, railway networks, communication networks, power networks, neural networks, and networks of unmanned cluster systems. Since the discovery of the small-world phenomenon and scale-free properties of complex networks in 1998 and 1999 [7–10], research in complex networks has emerged rapidly [11–13], becoming a new interdisciplinary field of broad interest. It has also gradually become a "powerful tool" for analyzing and researching complex systems, with significant research value and extensive application prospects [14–22].

Complex networks provide a powerful framework for modeling the interactions and relationships among entities in various systems, including those in human society, natural environments, and artificial systems. These networks serve as a foundation for studying the behaviors and dynamics of multi-agent systems. In particular, the application of reinforcement learning [23] in complex networks has opened new avenues for exploring how agents make decisions, adapt to their environments, and evolve their strategies over time. By leveraging the structural properties of complex networks, such as small-world and scale-free characteristics, reinforcement learning can be applied to analyze decision-making processes and cooperative dynamics in systems like social networks, communication networks, and unmanned swarm systems. This integration enables a deeper understanding of how network structure influences learning outcomes and cooperative behavior, making reinforcement learning a natural extension for studying the dynamics of complex networks. In recent years, many researchers have introduced reinforcement learning into evolutionary game studies, allowing individuals to adjust their decisions based on the payoffs from previous evolutionary rounds [24, 25], such as in the BM model [26]. As a simple reinforcement learning model based on expectations and historical payoffs, the BM model enables a population to maintain a certain level of cooperation even in cases of high social dilemma intensity and exhibit stronger cooperative behavior at appropriate expectation levels [27].

Inspired by the aforementioned theories, this paper proposes a coupled double-layer network structure that divides the network into two layers: a reinforcement layer and a traditional layer. The traditional layer consists of ordinary individuals using a conventional social learning model, while the reinforcement layer comprises strength individuals employing a modified BM model for reinforcement learning. These two network layers are not isolated; they are interconnected through a specific coupling mechanism. When making decisions, individuals are influenced not only by others within the same layer but also adjust their strategies based on information from the other layer. This mutual influence mechanism is designed to leverage the strengths of both learning approaches. The coupling of the two network layers leads to reciprocal influence during decision-making. Extensive experimental results indicate that in a coupled double-layer network, the overall cooperation level within the group increases significantly. Individuals in the traditional layer improve their behavior by imitating more successful strategies from the reinforcement layer. Due to the interaction between the two layers, strategy diversity is enhanced, enabling the group to adapt more effectively to environmental changes. The system’s resistance to external disturbances and internal fluctuations is strengthened, demonstrating greater robustness.

The remainder of the paper is organized as follows: Section 2 provides a detailed description of the model, Section 3 presents an in-depth analysis of the experimental results, and Section 4 summarizes the work conducted.

## 2. Model

The Prisoner’s Dilemma is introduced as the game model used by individuals, where each individual has two choices: cooperative behavior (*C*) and defection behavior (*D*). Depending on the choices made by individuals, they receive different payoffs. The standard payoff matrix used to represent these payoffs is as follows:

$\begin{array}{cc} & \begin{array}{cc} C & D \end{array}\\ \begin{array}{c} C\\ D \end{array} & \left(\begin{array}{cc} R & S\\ T & P \end{array}\right) \end{array}$ To reduce complexity, we adopt a simplified version of the Prisoner’s Dilemma model, setting the parameters as follows: R = 1, P = 0, and S = 0. The parameter T = b (where 1 ≤b≤2) is treated as a variable, representing the temptation to defect under different intensities of social dilemmas.

**Fig 1:**
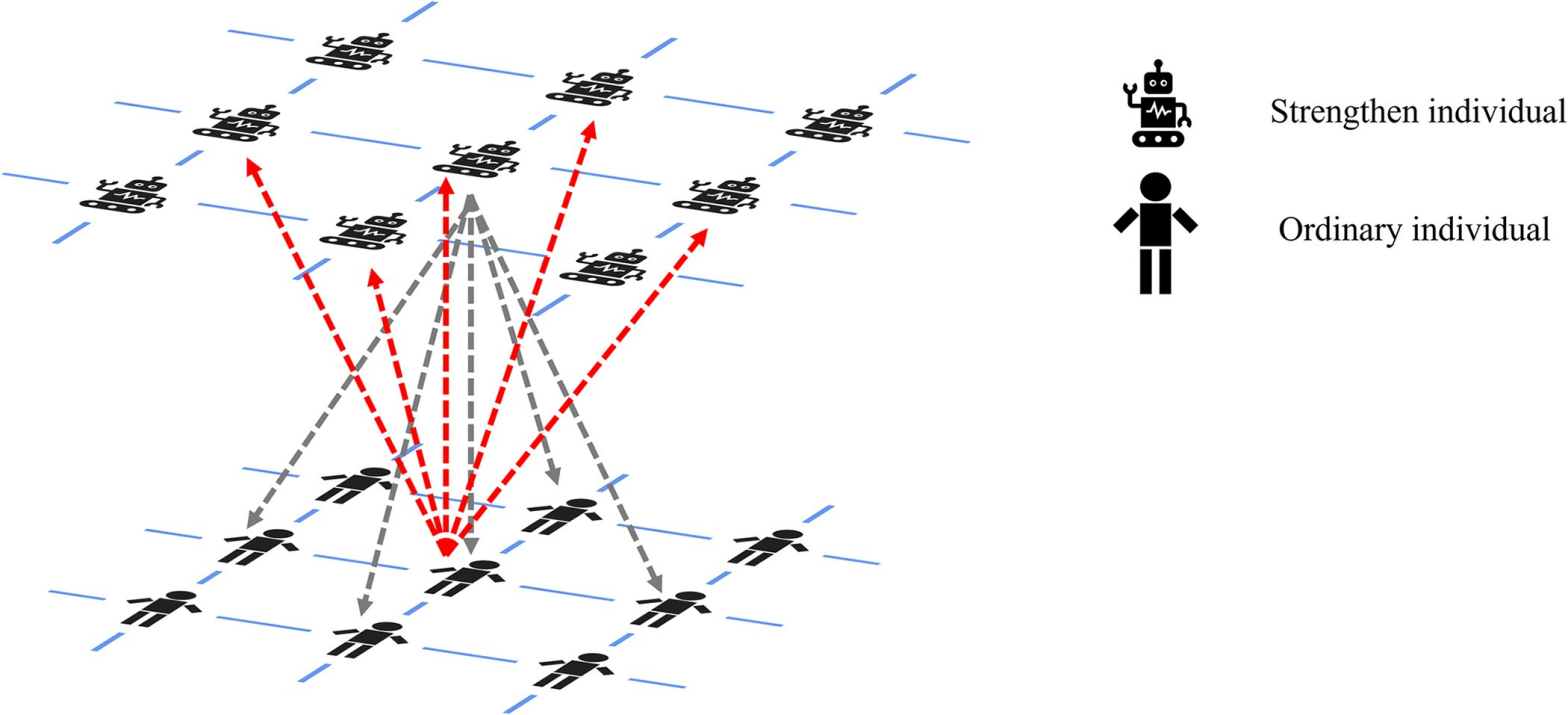
The specific structure of the model. Strength individuals update their strategies using the BM model based on reinforcement learning, while ordinary individuals update their strategies using social learning based on the Fermi rule. Individuals in the two layers can mutually influence each other. The gray lines represent the influence of strength individuals on ordinary individuals and their neighbors. The red lines indicate the influence of ordinary individuals on strength individuals and their neighbors.

The structure of the model is shown in Fig 1. In the network, ordinary individuals update their strategy choices using social learning based on the Fermi update rule. Since ordinary individuals are influenced by strength individuals, we have improved the calculation of payoffs for ordinary individuals by defining a composite payoff to replace the traditional concept of payoff. The specific calculation formula is as follows:

$$F_{x}=\alpha *P_{up}+(1-\alpha )*P_{x}$$ (1)

Here, *α* represents the degree of influence that strength individuals exert on ordinary individuals (where (0≤*α*≤1); a larger value of *α* indicates a greater influence. *P*_*up*_ is the payoff of the influencing strength individuals, which reflects this influence. Correspondingly, when ordinary individuals engage in social learning, they need to calculate their strategies using the following modified Fermi function:

$$W=\frac{1}{1+\exp\left[\left(F_{x}-F_{y}\right)/K\right]}$$ (2)

Here, *F*_*x*_ represents the composite payoff of the current individual *x*, and *F*_*y*_ represents the composite payoff of the neighboring individual *y* of individual *x*. *K* is a parameter used to indicate the noise intensity and irrational behavior. To maintain generality, we set *K* = 0.1 in this study, consistent with previous research [28, 29].

For strength individuals, who are intelligent agents generated through the integration of reinforcement learning techniques, they use a variant of the BM model to determine the probability of cooperating in the current round based on the probability of choosing cooperation in the previous round. This strategy update method allows strength individuals to make decisions based on their own experiences through reinforcement learning, rather than relying on their neighbors. In the t-th round of evolution, the calculation formula for the probability of an individual choosing to cooperate is as follows:

$$p_{t}=\{\begin{array}{ll} p_{t-1}+(1-p_{t-1})s_{t-1} & (a_{t-1}=C,s_{t-1}\geq 0),\\ p_{t-1}+p_{t-1}s_{t-1} & \left(a_{t-1}=C,s_{t-1}<0\right)\\ p_{t-1}-p_{t-1}s_{t-1} & \left(a_{t-1}=D,s_{t-1}\geq 0\right)\\ p_{t-1}-(1-p_{t-1})s_{t-1} & \left(a_{t-1}=D,s_{t-1}<0\right) \end{array}$$ (3)

Here, *p*_*t*−1_ represents the probability of the individual choosing to cooperate in the *t*−1-th round. *a*_*t*−1_∈(*C*, *D*) denotes the strategy chosen by the individual in the *t*−1-th round. *s*_*t*−1_ represents the stimulus signal that drives learning. If the individual is satisfied with the current payoff (*s*_*t*−1_ ≥ 0), indicating that the average payoff exceeds the expected value), the individual receives a positive stimulus; otherwise (*s*_*t*−1_ < 0), a negative stimulus is received. The calculation formulas are as follows:

$$s_{t-1}=\tanh\left[\beta (r_{t-1}-A)\right]$$ (4)

Here, *A* represents the expected payoff for strength individuals. *r*_*t*−1_ denotes the average composite payoff of the individual in the *t*−1-th round. The calculation method for the composite payoff here is similar to that in Formula 1. The specific calculation formula is as follows:

$$r_{t-1}=\left[\alpha *P_{down}+(1-\alpha )*P_{x}\right]/4$$ (5)

Here, the definition of *α* is the same as in Formula 1, and *P*_*down*_ is the payoff of the influencing ordinary individuals, which reflects this influence. In Formula 4, if *r*_*t*−1_−*A*>0, it indicates that the individual’s payoff in the *t*−1-th round exceeded the expected level, meaning the individual holds a positive attitude toward the strategy choice made in the *t*−1-th round. The parameter *β* represents the sensitivity of the difference between the payoff and the expectation in converting into a stimulus signal.

In each round of evolution, the strength individuals in the upper layer first undergo independent evolution. A strength individual is randomly selected to perform a Prisoner’s Dilemma Game (PDG), after which the probability of choosing cooperation is calculated using the reinforcement learning-based BM model, allowing for corresponding adjustments to the strategy. Following this, the ordinary individuals in the lower layer evolve under the influence of the strength individuals. A random ordinary individual is selected in the lower network to engage in a PDG with surrounding neighbors to obtain the corresponding payoffs. Then, the composite payoff of the individual is calculated according to the formulas, and finally, the strategy is updated using social learning based on the Fermi rule. Depending on the varying distance relationships between strength individuals and ordinary individuals, the degree of influence on ordinary individuals differs. This process is repeated in both the upper and lower layers for *L*×*L* iterations to ensure that each individual receives an update once during a complete Monte Carlo simulation step. A total of 10^4^ Monte Carlo simulation steps were conducted for the experiments. The results were obtained by averaging the generated values over the last 2000 steps for further analysis, ensuring the accuracy of the simulations.

## 3. Result

**Fig 2:**
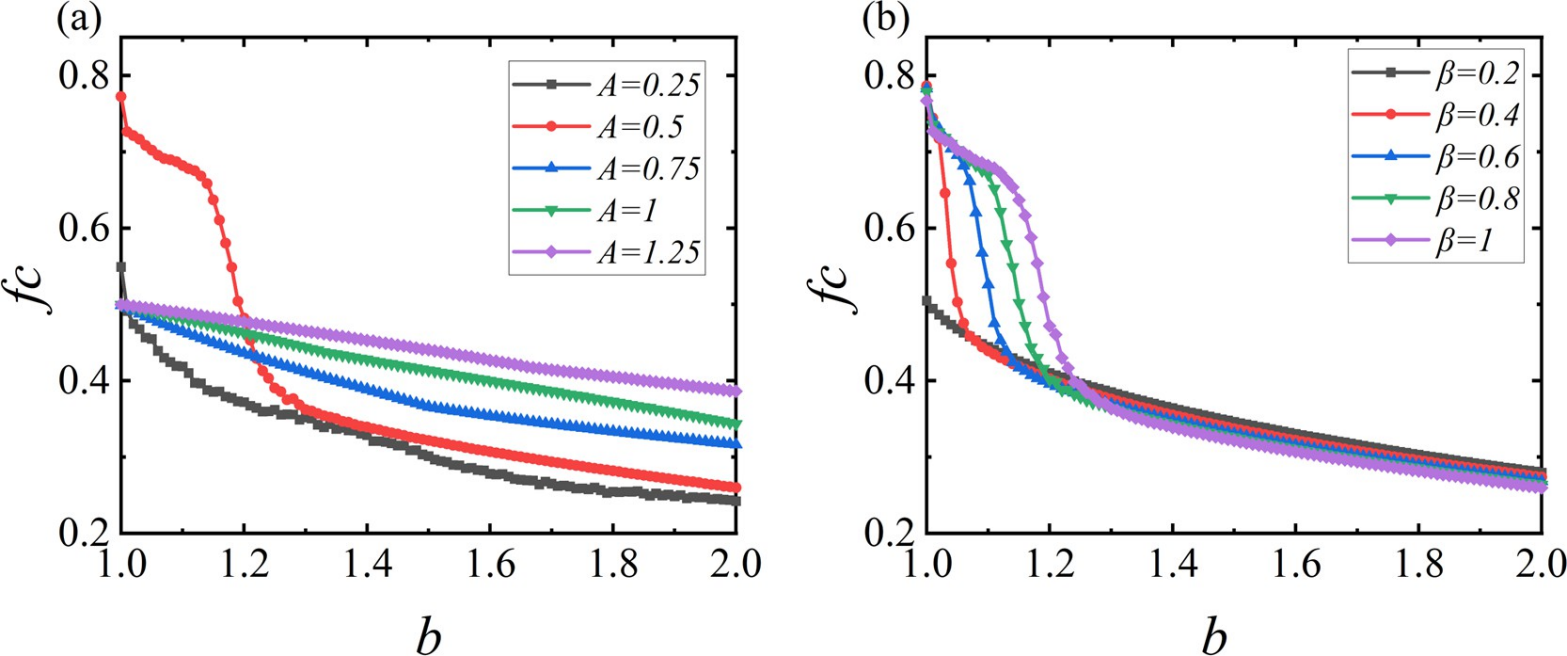
Appropriate expectations and higher sensitivity can enhance the cooperative behavior of strength individuals. The influence of the two parameters on the cooperation rate *fc* of strength individuals under different levels of temptation to defect b is shown. (a) The impact of the expected level *A* on the cooperation rate of strength individuals. (b) The effect of the sensitivity parameter *β* on the cooperation rate of strength individuals. Here, *L* is set to 200.

We first analyze the introduced strength individuals. According to Formulas 3 and 4, the performance of the strength individuals is influenced not only by their historical payoffs but also by another important factor, the stimulus signal *s*_*t*−1_, which drives learning and is determined by the sensitivity parameter *β* and the expected payoff *A*. Under different values of *b*, we obtained the effects of these two parameters on the cooperation rate of strength individuals, as shown in Fig 2. Simulation experiments indicate that the introduced strength individuals can maintain a certain level of cooperative behavior even under high social dilemmas. In Fig 2(A), we selected several different expected levels. Different values of *A* represent varying expected levels; a higher *A* indicates a higher expectation and vice versa. The experimental results show that the relationship between the expected level and the group’s cooperative performance is quite complex and not a simple linear relationship. Both excessively high and low expected levels can impact the emergence and maintenance of cooperative behavior. Extreme expected levels create a significant gap between actual payoffs and expectations, making it difficult to sustain cooperative behavior, leading to a decrease in the cooperation rate. Regarding the other parameter *β* in Formula 4, which represents the stimulus signal driving learning, we selected several different sensitivity levels in Fig 2(B). The *β* values range from low to high sensitivity. From the figure, we can see that the sensitivity parameter *β* is positively correlated with the group’s cooperative performance; as *β* increases, the cooperative performance of the strength individuals improves. A higher sensitivity means that strength individuals are more attuned to the discrepancies between actual payoffs and expectations, allowing them to quickly recognize the gaps caused by their current strategies and adjust accordingly. This enables strength individuals to promptly revise their suboptimal strategy choices and select better strategies, thereby increasing the group’s cooperation rate. Therefore, in subsequent studies, we will fix the expected level *A* at the optimal value of 0.5 and set the sensitivity parameter *β* to 1 to obtain better results.

**Fig 3:**
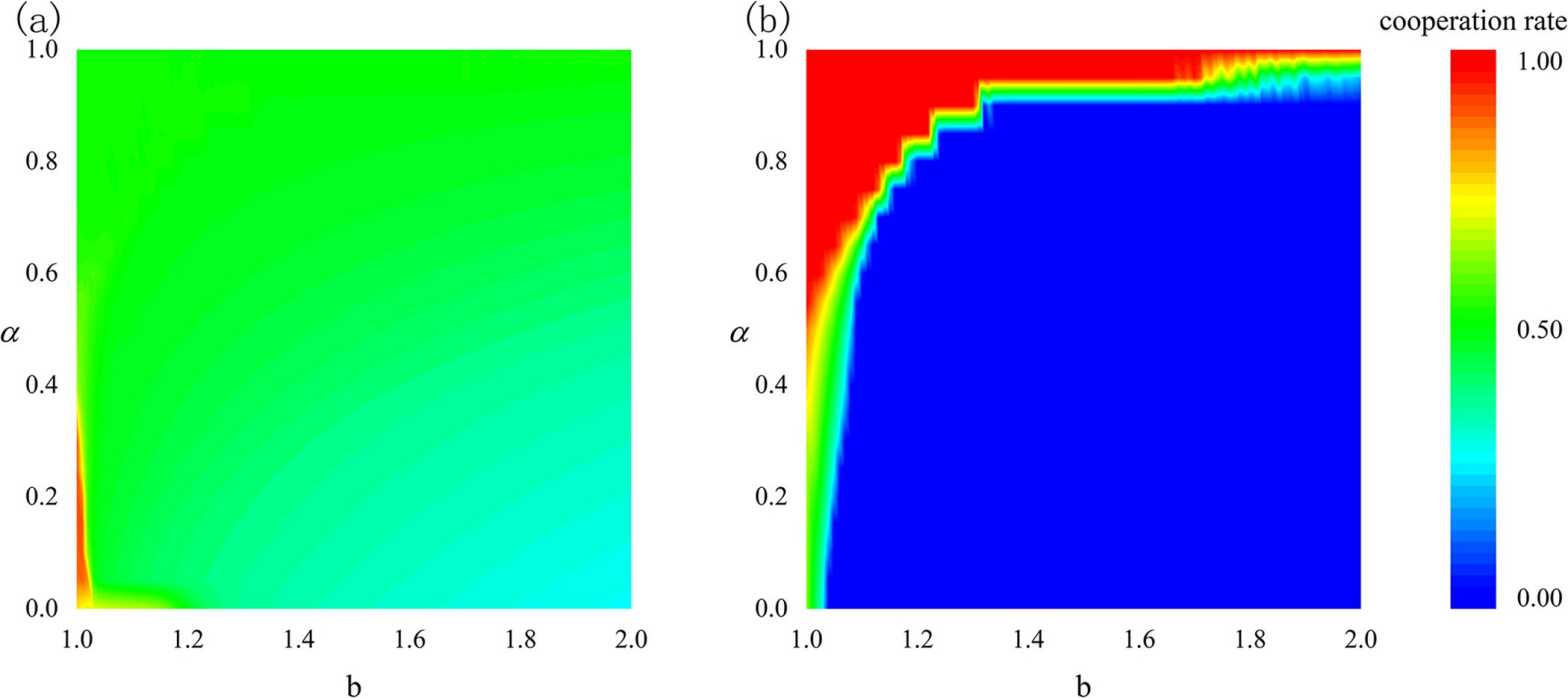
The effects of the influence degree *α* and the temptation to defect *b* on the cooperation rate of the groups. (a) Changes in the cooperation rate of strength individuals; (b) Changes in the cooperation rate of ordinary individuals. Here, *L* is set to 200.

In the network, strength individuals in the upper layer influence the ordinary individuals in the lower layer, and conversely, the ordinary individuals in the lower layer can also affect the strength individuals. Fig 3 illustrates the effects of the influence degree *α* and the temptation to defect *b* on the cooperation rate of the groups. A larger *α* indicates that the proportion of upper-layer strength individuals in the calculation of the composite payoff for the lower-layer ordinary individuals is greater. The same applies to the upper-layer strength individuals. Fig 3(A) shows the results of the cooperation rate of strength individuals as *α* and *b* vary. From the figure, it can be observed that with a low *α* (close to 0), when *b* is small (close to 1.0), the group’s cooperation rate is high. However, as the influence degree *α* increases, the cooperation rate decreases and eventually stabilizes, gradually transitioning into the green and cyan areas. When the group responds more strongly to cooperation signals, the cooperation rate can be maintained at a high level even under high temptation to defect. Furthermore, although the cooperation rate of strength individuals shows a slight decline as *b* increases, the overall change is small, remaining at a high level. This indicates that strength individuals still possess a strong cooperative capability when facing high temptation to defect. Fig 3(B) displays the results of the cooperation rate of ordinary individuals as *α* and *b* vary. When *α* is high (close to 1.0), the group can still maintain a high cooperation rate even with a large temptation to defect *b*. However, as *α* decreases and the temptation to defect *b* increases, the cooperation rate of the group drops sharply, gradually entering the blue area, which indicates a cooperation rate close to 0. This suggests that ordinary individuals are highly vulnerable to cooperation when facing temptation to defect; once the payoff from defection exceeds a certain threshold, maintaining cooperative behavior becomes difficult. The experimental results reveal significant differences in cooperation rates between the two types of groups. Strength individuals can maintain a high cooperation rate even under high temptation to defect, possibly because this group is more sensitive to cooperation signals, possesses stronger cooperative mechanisms, and has better information-sharing capabilities. In contrast, ordinary individuals can maintain a high cooperation rate under low temptation to defect, but when the temptation exceeds a certain critical value, the cooperation rate declines sharply. This indicates that ordinary individuals’ cooperative behavior easily collapses when faced with high temptation to defect. Theoretically, strength individuals may possess greater adaptability and resilience, allowing them to self-regulate when confronted with varying degrees of temptation to defect, whereas ordinary individuals lack this resilience.

**Fig 4:**
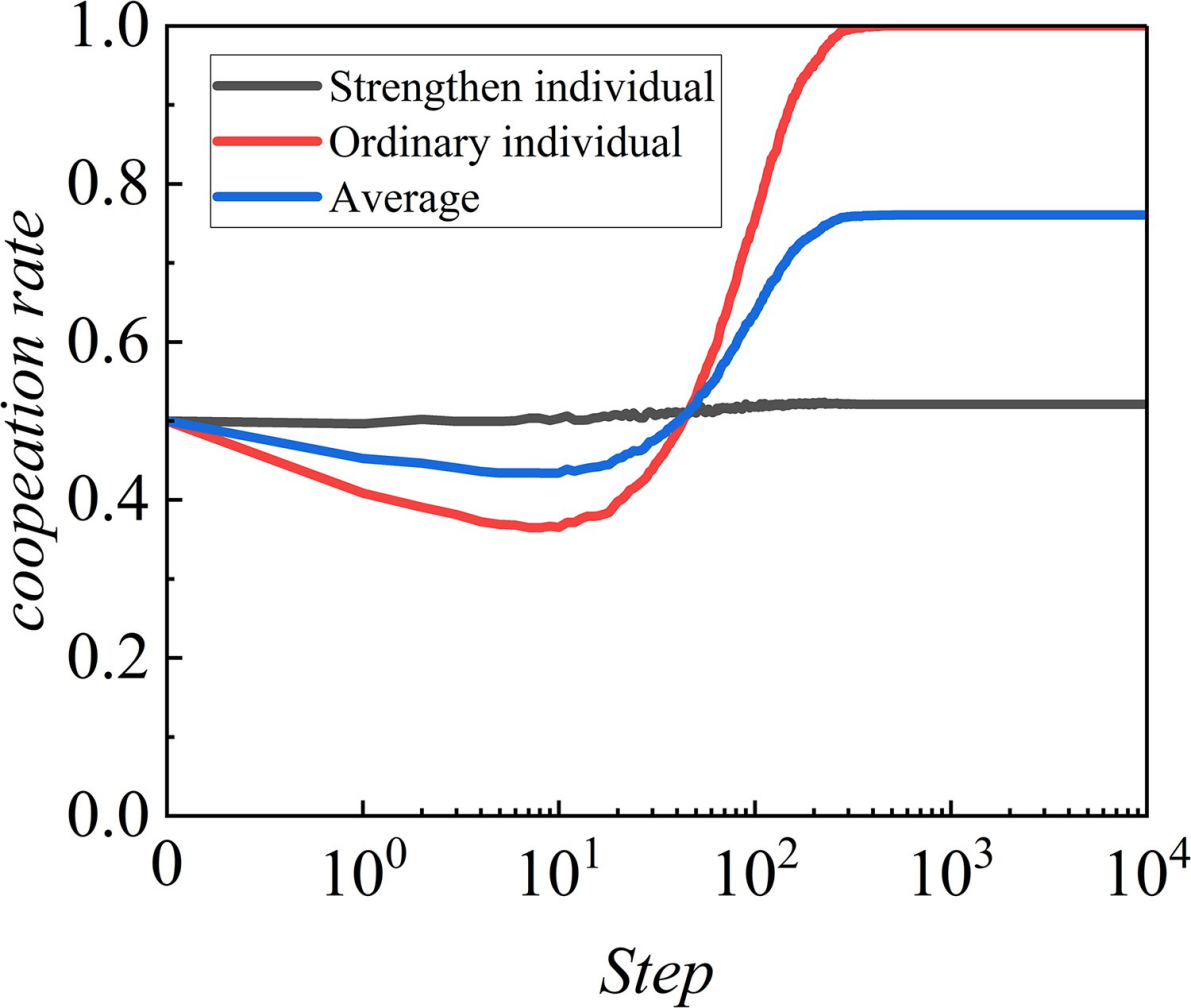
The change trends of cooperation rates for different types of groups (strength individuals and ordinary individuals) as the step length increases. Here, *L* is set to 200, *b* is set to 1.04, and *α* is set to 0.8.

Fig 4 shows the trend of cooperation rates in different groups as the step length varies, where the cooperation rate reflects the level of collaboration among individuals within the group, and the step length represents the time progression of the system’s evolution. The strength individuals exhibit a highly stable cooperation rate throughout the entire range of step lengths. The initial cooperation rate is set at 50%, and during the progression from the beginning to the end of the step length, there are virtually no significant fluctuations or declines. This indicates that strength individuals possess a strong cooperative mechanism or that the algorithm design emphasizes collaboration among individuals. Even as the step length increases, the cooperative relationships among individuals remain stable, and cooperative behavior is not significantly disrupted by external factors. This phenomenon suggests that this group has high robustness and resilience against defection. In contrast, the cooperation rate of ordinary individuals displays a different trend. Initially, the cooperation rate rapidly declines from 50% to a low of nearly 30%, indicating that in the early stages of the system, the cooperative relationships among ordinary individuals are relatively fragile and easily undermined by temptation to defect. This may relate to the decision rules individuals employ when making cooperation choices. In the early stage, ordinary individuals may be particularly sensitive to the payoffs from defection, leading to an overall decrease in cooperation rate. However, as the step length increases, around 10^1^ steps, the cooperation rate of ordinary individuals experiences a significant rebound and rises rapidly, ultimately surpassing that of the strength individuals, reaching nearly 100%. This change indicates that after experiencing early cooperative dilemmas, ordinary individuals gradually establish effective cooperation mechanisms. In the later stages of the system, this group displays even more proactive cooperation behavior, exceeding that of strength individuals. The trend of this curve reveals the adaptability of ordinary individuals over a longer time scale. It appears that ordinary individuals require more time to explore and discover stable cooperation; once a stable cooperative relationship is established, their level of cooperation significantly improves and maintains a high level.

**Fig 5:**
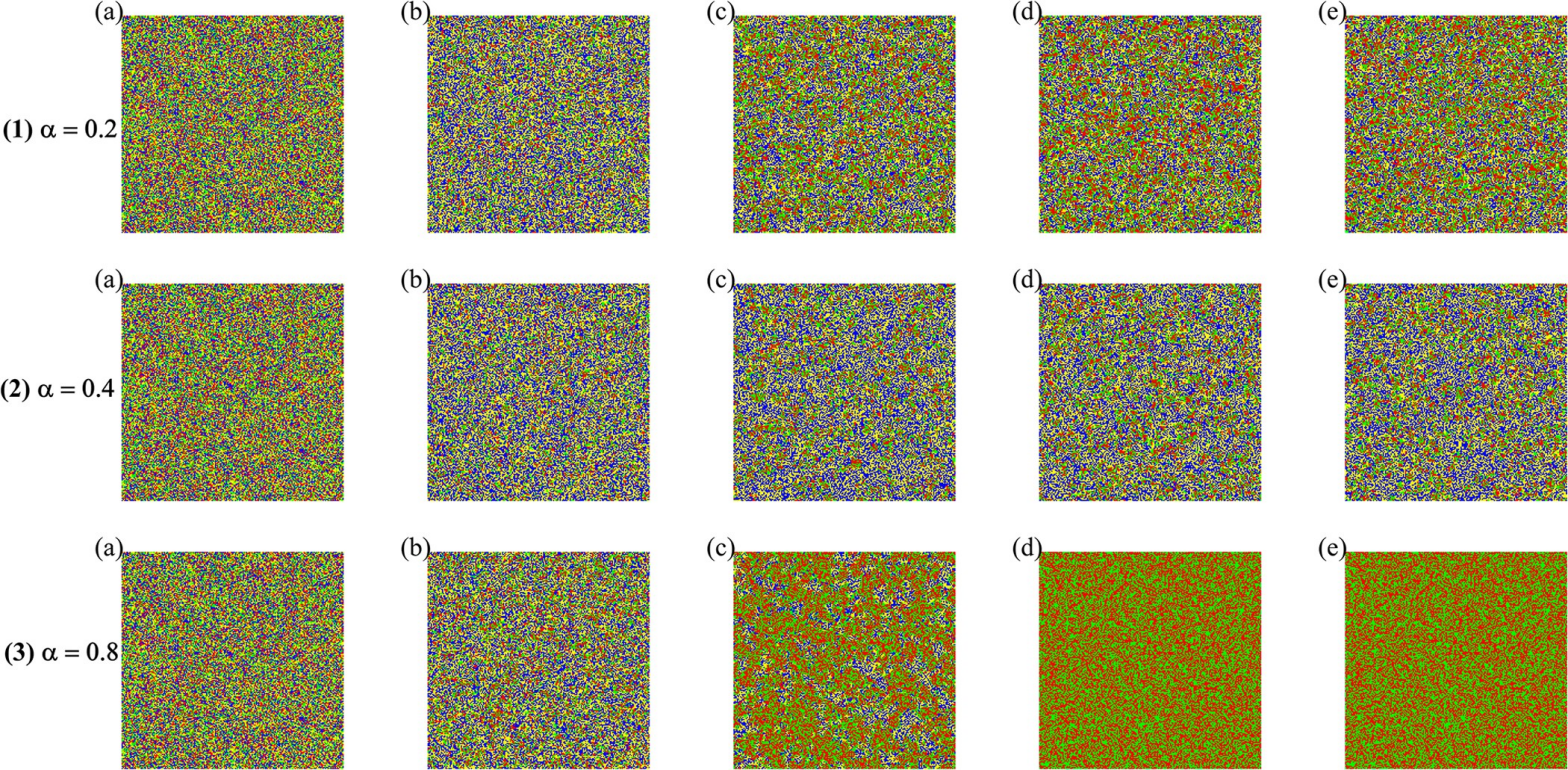
Changes in strategy interaction pairs within the group under different conditions of *α*. The colors represent the following strategies: red for CC (both strength and ordinary individuals choosing cooperation), yellow for CD (strength individuals cooperating while ordinary individuals betray), blue for DD (both groups betraying), and green for DC (strength individuals betraying while ordinary individuals cooperate). The steps are represented from left to right (a-e) corresponding to 0, 5, 100, 1000, and 10,000. Here, *L* is set to 200, and *b* is set to 1.04.

In the double-layer network, to study the interactions between strength individuals and ordinary individuals, we defined four types of interaction strategy pairs based on the strategy choices of the strength and ordinary individuals: both strength and ordinary individuals choose cooperation (CC); strength individuals choose cooperation while ordinary individuals choose betrayal (CD); strength individuals choose betrayal while ordinary individuals choose cooperation (DC); and both strength and ordinary individuals choose betrayal (DD). Using these four interaction strategy pairs, we obtained the changes in the interactions within the group during the evolutionary process, as shown in Fig 5. From the figure, it can be observed that when *α* = 0.8, the cooperation level of the group reaches its peak, which is consistent with the optimal value in Fig 3(B). In the final stage of evolution, CC became the dominant interaction strategy among the four pairs. This phenomenon indicates that the strength individuals, by choosing cooperation, successfully influenced the ordinary individuals in the lower layer, leading them and their surrounding neighbors to also adopt cooperative strategies. At the same time, we can see that during the evolutionary process, there is a clustering phenomenon among the betraying ordinary individuals. This is related to the fact that ordinary individuals employ social learning based on the Fermi rule for strategy learning. If no intervention is made regarding these betrayal clusters, these ordinary individuals will fall into a betrayal dilemma from which they cannot escape. However, in the dual-layer network, the intervention of strength individuals in the upper layer enables the ordinary individuals in these betrayal clusters to successfully escape the dilemma and instead choose cooperative strategies.

In the context of higher *α*, ordinary individuals who cooperate gain more benefits than those who defect due to several interconnected factors. First, a higher *α* increases the influence of strength individuals’ payoffs on the composite payoffs of ordinary individuals. Ordinary individuals that cooperate (CC) with cooperative strength individuals benefit significantly from this interaction, as cooperation creates a stable and mutually beneficial environment. On the contrary, defectors (CD or DC) reduce the overall system stability, leading to lower cumulative payoffs. Second, as seen in the figure, higher *α* promotes the dominance of cooperative strategies over time, evident from the increasing red regions (CC) as the steps progress, especially at (*α* = 0.8). This demonstrates that cooperative strategies yield higher long-term payoffs, driving the system toward a cooperation-dominated equilibrium. Lastly, the interaction dynamics indicate that cooperative behavior among ordinary individuals fosters a more favorable environment for strength individuals, who, in turn, reinforce cooperation through their influence on ordinary individuals’ payoffs. Defectors, on the other hand, fail to adapt to this cooperative environment, resulting in lower payoffs and eventual decline. Thus, higher *α* aligns the system dynamics to favor cooperative strategies, leading to higher rewards for cooperative ordinary individuals.

## 4. Conclusion

This article investigates the evolution of cooperation in hybrid collectives by proposing a method based on a dual-layer network structure, where reinforcement agents are distributed in the upper network and can influence ordinary individuals in the lower network. Conversely, ordinary individuals can also impact reinforcement agents. Within this framework, reinforcement agents transmit cooperation signals or strategies to ordinary individuals through their strategy selections and decision rules, thereby affecting the overall level of cooperation in the collective. This dual-layer network structure not only simulates the strategy evolution in complex collectives but also demonstrates the collaborative dynamics between different types of individuals, especially in response to varying choices between cooperation and betrayal. To validate the effectiveness of the model, we conducted extensive simulations using the Monte Carlo method. The experiments simulated the cooperative evolution of the collective under different levels of betrayal temptation and influence intensity. The results indicate that the introduction of reinforcement agents in the dual-layer network significantly enhances the overall cooperative performance of the group. Under low betrayal temptation, reinforcement agents can effectively maintain a high level of cooperation within the collective, as ordinary individuals tend to follow the cooperative strategies of the reinforcement agents, even when faced with some pressure to betray. In contrast, even under high betrayal temptation, where the allure of betrayal increases, cooperation behaviors within the group can still be upheld, preventing a complete collapse into betrayal. This underscores the critical role that reinforcement agents play in sustaining cooperation within the group. We hope that the proposed method can provide valuable insights for future research. The application of AI technology is continuously expanding, especially in the field of collective coordination. How to enhance collective efficiency through interactions among agents has become an important issue that needs to be addressed. By combining a dual-layer network structure with reinforcement learning methods, we demonstrate the potential for maintaining efficient cooperation in complex environments. This not only helps solve the problem of cooperative evolution in collectives but also promotes the deep application of AI technology in intelligent collectives and multi-agent systems.

## Supporting information

S1 Data This is the data of this paper. (ZIP)
